# Quantitative mitral regurgitation and conventional echocardiographic variables in canine myxomatous mitral valve disease

**DOI:** 10.3389/fvets.2026.1885087

**Published:** 2026-07-03

**Authors:** Shimpei Kawai, Ryohei Suzuki, Yohei Mochizuki, Yunosuke Yuchi, Shuji Satomi, Arata Kitazawa, Masanobu Teraoka, Takahiro Teshima, Hirotaka Matsumoto

**Affiliations:** 1 Laboratory of Veterinary Internal Medicine, Faculty of Veterinary Medicine, School of Veterinary Science, Nippon Veterinary and Life Science University, Musashino, Tokyo, Japan; 2 Faculty of Veterinary Medicine, Okayama University of Science, Imabari, Ehime, Japan

**Keywords:** Doppler imaging, heart failure, regurgitant fraction, regurgitant volume, velocity-time integral

## Abstract

**Introduction:**

Disease severity in dogs with myxomatous mitral valve disease (MMVD) is commonly assessed using conventional echocardiographic variables and staging systems based on cardiac remodeling and heart failure status. Multiparametric scoring systems incorporating variables such as early diastolic transmitral velocity (E-wave) have also been recently proposed. However, these approaches primarily reflect secondary morphological and loading changes rather than directly quantifying severity of mitral regurgitation (MR). Therefore, quantitative regurgitant indices may provide additional information regarding the functional severity of MR.

**Methods:**

This retrospective study included 167 predominantly small-breed dogs with MMVD evaluated at Japanese referral centers, classified according to the American College of Veterinary Internal Medicine (ACVIM) staging system. Body weight–normalized regurgitant volume (RVol/kg) and regurgitant fraction (RF) were calculated using volumetric methods. Group comparisons among ACVIM stages, correlation analysis, logistic regression analysis, and receiver operating characteristic analysis were performed to evaluate associations between regurgitant indices and conventional echocardiographic variables.

**Results:**

RVol/kg and RF increased significantly with advancing disease stage (both *p* < 0.001). However, no significant differences were observed between stages B2 and C/D and severe MR (RF ≥ 50%) was identified in some dogs classified as stage B1. Quantitative regurgitant indices showed significant correlations with the left atrium-to-aorta ratio, body-weight–normalized left ventricular internal diameter in diastole (LVIDDN), and E-wave, whereas correlations with fractional shortening were weak. The mitral-aortic velocity-time integral (VTI) ratio demonstrated the strongest correlations with both RVol/kg and RF (both *r* = 0.75; *p* < 0.001) and showed the highest diagnostic performance in identifying severe MR (area under the curve = 0.89). In the multivariable analysis, LVIDDN and the mitral-aortic VTI ratio remained independent predictors of severe MR.

**Discussion:**

Quantitative regurgitant indices were associated with conventional echocardiographic variables and may provide complementary information regarding MR severity in dogs with MMVD. In this predominantly small-breed Japanese MMVD population, the mitral-aortic VTI ratio showed strong associations with quantitative regurgitant indices and may be a clinically useful Doppler-derived surrogate for MR severity, although prospective studies are required to confirm these findings.

## 1 Introduction

Myxomatous mitral valve disease (MMVD) (1), the most common acquired cardiac disease in dogs, is characterized by progressive myxomatous degeneration of the mitral valve apparatus, resulting in mitral regurgitation (MR). Chronic MR leads to volume overload in the left atrium and left ventricle, causing cardiac enlargement, increased filling pressure, and ventricular remodeling. These hemodynamic changes are major contributors to the development of congestive heart failure (CHF) and are closely associated with the prognosis. Therefore, accurate assessment of MR severity is essential for understanding disease status and guiding clinical management in dogs with MMVD.

Currently, disease severity in MMVD is commonly assessed using the American College of Veterinary Internal Medicine (ACVIM) staging system (2), which classifies patients based on cardiac enlargement and clinical signs of heart failure. Recently, multiparametric scoring systems (3, 4) incorporating conventional echocardiographic variables, including early diastolic transmitral velocity (E-wave), have also been proposed. These approaches integrate morphological and Doppler-derived indices, including the left atrium-to-aorta (LA/Ao) ratio, body-weight–normalized left ventricular internal diameter in diastole (LVIDDN), E-wave, and fractional shortening (FS) to evaluate disease progression. However, these indices primarily reflect the structural remodeling and hemodynamic consequences of MR rather than directly quantifying the regurgitation itself. Consequently, they may not fully capture the underlying severity of MR.

Furthermore, individual echocardiographic parameters reflect different aspects of the disease process, such as volume overload, filling pressure, and ventricular remodeling, and do not necessarily change simultaneously. Therefore, discrepancies among these indices can occur, making it difficult to comprehensively assess MR severity using a single parameter. This highlights a clinical limitation of current evaluation strategies in which morphological and functional indices may not fully align (5) with the underlying hemodynamic burden of MR.

In human cardiology, quantitative assessment of MR using regurgitant volume (RVol) and regurgitant fraction (RF) is widely accepted for evaluating disease severity (6). These parameters are typically derived from volumetric methods and are used to define severe MR (e.g., RF ≥ 50%) (6, 7). Although similar approaches may be applicable in veterinary medicine, their use in routine clinical practice remains limited because of the complexity of the required measurements. Therefore, it is necessary to evaluate the clinical utility of quantitative MR indices that directly reflect the hemodynamic burden of regurgitation.

In addition, the mitral-aortic velocity-time integral (VTI) ratio has recently been proposed as a simple Doppler-derived surrogate for MR severity (8–11). This index does not require geometric assumptions and can be obtained rapidly, making it suitable for clinical use. A recent large-scale study involving 1,109 dogs with MMVD demonstrated significant associations of the mitral-aortic VTI ratio with RF, conventional echocardiographic variables, and ACVIM stage. The study also reported good diagnostic performance for identifying severe MR (11). Another study involving 237 dogs with MMVD used the left ventricular early inflow-outflow index, a Doppler-derived index conceptually related to transmitral flow normalized to forward outflow, and systematically examined the relationships between volumetrically derived RF and conventional echocardiographic variables (12). Together, these studies support the clinical utility of quantitative and Doppler-derived approaches for assessment of MR severity in dogs with MMVD. However, these previous studies were conducted primarily in European populations with broader breed distributions. In contrast, MMVD in Japan predominantly affects small-breed dogs. Because quantitative assessment of MR and calculation of the mitral-aortic VTI ratio rely on measurements such as left ventricular outflow tract (LVOT) diameter and Doppler-derived VTI measurements, their applicability in very small-breed dogs cannot be assumed. To our knowledge, validation of the mitral-aortic VTI ratio in a predominantly small-breed Japanese MMVD population has not been previously reported. Therefore, further evaluation in such a population is warranted.

This study aimed to evaluate the applicability and clinical utility of the mitral-aortic VTI ratio in a predominantly small-breed Japanese MMVD population by examining its relationship with quantitative MR indices (RVol and RF) and conventional echocardiographic variables. In addition, we compared the diagnostic performance of the mitral-aortic VTI ratio with commonly used conventional echocardiographic variables for identifying severe MR based on established human criteria and assessed whether combining these variables altered diagnostic performance.

## 2 Materials and methods

### 2.1 Ethics statement

This retrospective cohort study was approved by the Ethics Committee of the Nippon Veterinary and Life Science University (Approval numbers: R2-5 and 25–07). All the animal owners provided written informed consent to participate in the study.

### 2.2 Study design and animals

Client-owned dogs diagnosed with MMVD between April 1, 2023, and July 31, 2024, at Nippon Veterinary and Life Science University Veterinary Medical Center and Okayama University of Science Veterinary Teaching Hospital were retrospectively included.

The diagnosis of MMVD was based on characteristic echocardiographic findings, including thickening and/or prolapse of the mitral valve leaflets, accompanied by MR identified by color Doppler imaging. Diagnosis was confirmed using a comprehensive echocardiographic examination, including two-dimensional, color Doppler, pulsed-wave Doppler, and continuous-wave Doppler modalities (13, 14).

Dogs with congenital heart disease, mitral valve dysplasia, infective endocarditis, cardiomyopathy, or atrial fibrillation were excluded. Mitral valve dysplasia and infective endocarditis were excluded based on signalment, clinical history, physical examination findings, and comprehensive echocardiographic assessment. Dogs with high-velocity turbulence in the LVOT or inadequate echocardiographic image quality were also excluded. Only one examination per dog was included in the study. When multiple echocardiographic examinations were available for the same dog, only the first examination meeting the inclusion criteria was analyzed.

### 2.3 Classification

Dogs were classified into ACVIM stages B1, B2, and C/D according to the 2019 consensus guidelines (2) based on clinical signs, thoracic radiographic findings, echocardiographic evidence of cardiac remodeling, and history of CHF. Age, sex, and body weight were recorded. When available, the treatment history and prior diagnostic results were reviewed.

### 2.4 Conventional echocardiography examination

Echocardiographic examinations were performed using a phased-array transducer-equipped ultrasound system (Vivid E95; GE Healthcare, Tokyo, Japan). Stored images were analyzed retrospectively. No dogs were sedated during echocardiographic examination.

Simultaneous lead II electrocardiographic recordings were obtained. Sinus rhythm was verified using the simultaneously recorded electrocardiographic tracings, and measurements were averaged over three consecutive cardiac cycles during sinus rhythm. To minimize the respiratory influence, cardiac cycles with minimal thoracic motion were selected.

Standard right parasternal and left apical views (15, 16) were obtained, and offline analyses were performed using dedicated software (EchoPAC version 204; GE Healthcare).

The LA/Ao ratio was measured from the right parasternal short-axis view at the level of the aortic valve during early diastole (17). LVIDDN was measured at end-diastole from the right parasternal short-axis view at the chordae tendineae level, primarily using M-mode recordings. When optimal cursor alignment perpendicular to the interventricular septum and left ventricular free wall could not be achieved, measurements were obtained from two-dimensional images. LVIDDN was calculated according to a previously described allometric scaling method, and agreement between M-mode and two-dimensional measurements obtained from the same imaging plane has been previously reported (18, 19). FS was calculated from the left ventricular internal diameters obtained at end-diastole and end-systole in the same imaging plane. E wave was obtained using pulsed-wave Doppler from the left apical four-chamber view, with the sample volume positioned at the mitral leaflet tips.

### 2.5 Measurement of regurgitant indices

Left ventricular end-diastolic and end-systolic volumes were measured using the modified Simpson’s method of disks from the left apical four-chamber view (20–22). End-diastole was defined as the onset of the QRS complex or maximal left ventricular dimension, whereas end-systole was defined as the minimal left ventricular dimension.

The total stroke volume (SV) was calculated by subtracting the end-systolic volume from the end-diastolic volume. The forward SV through the LVOT SV was calculated as the product of the LVOT cross-sectional area and LVOT VTI. The LVOT cross-sectional area was calculated using the following equation:

$$\text{LVOT cross}-\text{sectional area}=\pi \times {\left(L\mathrm{VOT}\;\text{diameter}/2\right)}^{2}.$$

The LVOT VTI was measured using pulsed-wave Doppler from the left apical five-chamber view, with the sample volume positioned immediately below the aortic valve at the subaortic LVOT level. The LVOT diameter was measured at the aortic annular level (hinge-point to hinge-point) in the right parasternal long-axis view during mid-systole, corresponding to maximal opening of the aortic valve leaflets. In accordance with published echocardiographic recommendations (6), this annular diameter measurement was used for calculation of LVOT cross-sectional area and forward SV. Representative echocardiographic measurements used for calculation of the quantitative regurgitant indices are shown in Supplementary Figure S1.

RVol was calculated by subtracting the LVOT SV from the total SV (6). The RF was calculated using the following equation:

RF(%)=(RVol/totalSV)×100.

To account for body size, RVol was indexed to body weight (RVol/kg) (23). Both RVol/kg and RF were used as primary quantitative regurgitant indices in subsequent analyses. Based on previous volumetric observations showing that RVol/kg values greater than 1.0 mL/kg were common in dogs with more advanced MMVD, RVol/kg = 1.0 mL/kg was used as a visual reference for graphical presentation (23). This value was not used for disease classification or for defining severe MR.

Transmitral inflow and LVOT VTIs were obtained from pulsed-wave Doppler tracings recorded from the left apical four- and five-chamber views, respectively (9, 11). The mitral-aortic VTI ratio was calculated as the transmitral inflow VTI divided by LVOT VTI. This Doppler-derived index was evaluated as a secondary parameter.

### 2.6 Statistical analyses

Statistical analyses were performed using R version 4.5.0 (The R Foundation for Statistical Computing, Vienna, Austria) and EZR version 1.68 (Jichi Medical University, Saitama Medical Center, Saitama, Japan). Continuous variables are presented as median (interquartile range). Normality was assessed using the Shapiro–Wilk test. Group comparisons were performed using one-way or Welch’s analysis of variance with Games-Howell *post hoc* testing for normally distributed data and the Kruskal-Wallis test with Steel-Dwass post hoc testing for non-normally distributed data. Associations between regurgitant indices (RVol/kg and RF) and conventional echocardiographic parameters were assessed using Spearman’s rank correlation coefficients. Severe MR was defined as RF ≥ 50%. Diagnostic performance was evaluated using receiver operating characteristic (ROC) curve analysis, and the area under the curve (AUC), optimal cutoff values, sensitivity, specificity, positive predictive values, and negative predictive values were calculated. Optimal cutoff values were determined using the Youden index. Logistic regression analysis was performed to identify predictors of severe MR. Variables showing statistical significance in the univariable analysis were included in the multivariable models. Collinearity was assessed using variance inflation factors, and multivariable models were constructed using backward/forward stepwise selection based on the Akaike information criterion. The results were expressed as odds ratios with 95% confidence intervals (CI). The combined models derived from the independent predictors were evaluated using ROC analysis. Comparisons between ROC curves were performed using DeLong’s test when appropriate. A value of *p* < 0.05 was considered statistically significant.

## 3 Results

### 3.1 Study population

Of 174 dogs that were initially included in this study, 7 were excluded (4 had high-velocity turbulence in the LVOT and 3 had inadequate image quality). Finally, 167 dogs were included in the analysis.

The study population comprised 83 dogs with stage B1 disease, 57 dogs with stage B2 disease, and 27 dogs with stage C/D disease. All dogs in the C/D group had current or previous CHF and were classified as having stage C. None of the dogs met the criteria for stage D.

The most common breeds were Chihuahua (32/167), Toy Poodle (27/167), Cavalier King Charles Spaniel (17/167), Pomeranian (16/167), Miniature Dachshund (14/167), and mixed-breed dogs (16/167). Detailed breed distribution according to ACVIM stage is provided in Supplementary Table S1. Treatment status according to ACVIM stage is summarized in Supplementary Table S2.

The baseline characteristics are summarized in Table 1. Age was significantly higher in patients with stage C/D than in those with stage B1 (*p* < 0.05), and body weight decreased with disease progression (*p* < 0.001).

**Table 1:** Baseline characteristics of the study group.

Variable	Stage B1 (n = 83)	Stage B2 (n = 57)	Stage C/D (n = 27)
Age (years)	11.3 (8.7, 12.6)	11.9 (10.1, 13.4)	12.5 (12.0, 13.6)^#^
Sex (female/male)	42/41	28/29	21/6
Body weight (kg)	5.5 (4.0, 7.7)	4.5 (3.1, 6.3)*	3.2 (2.9, 6.2)*
FS (%)	42.0 (35.1, 51.0)	51.5 (46.4, 59.6)^#^	55.7 (51.5, 61.4)^#^
LA/Ao	1.3 (1.1, 1.5)	1.9 (1.7, 2.2)^#^	2.1 (1.9, 2.5)^#^
LVIDDN	1.5 (1.3, 1.6)	1.8 (1.7, 2.0)^#^	1.9 (1.6, 2.4)^#^
E-wave (m/s)	0.73 (0.56, 0.85)	1.1 (0.89, 1.2)^#^	1.2 (1.0, 1.3)^#^
RVol/kg (mL/kg)	0.26 (0.02, 0.51)	1.2 (0.74, 1.8)^#^	1.6 (1.2, 2.2)^#^
RF (%)	13.5 (1.9, 28.3)	53.3 (38.7, 65.8)^#^	60.2 (52.4, 72.1)^#^
Mitral–aortic VTI ratio	0.98 (0.88, 1.2)	1.5 (1.2, 1.9)^#^	1.7 (1.5, 2.3)^#^

Values are presented as medians (interquartile range) or n/n.

*p < 0.05 vs. B1; ^#^*p* < 0.001 vs. B1.

E-wave, early diastolic transmitral velocity; FS, fractional shortening; LA/Ao, left atrium-to-aorta ratio; LVIDDN, body-weight–normalized left ventricular internal diameter in diastole; RF, regurgitant fraction; RVol/kg, regurgitant volume normalized to body weight; VTI, velocity-time integral.

### 3.2 Conventional echocardiographic parameters across ACVIM stages

Conventional echocardiographic variables are summarized in Table 1. The LA/Ao ratio, LVIDDN, E-wave, and FS increased significantly with advancing stage (all *p* < 0.001).

### 3.3 Regurgitant indices across ACVIM stages

**Figure 1:**
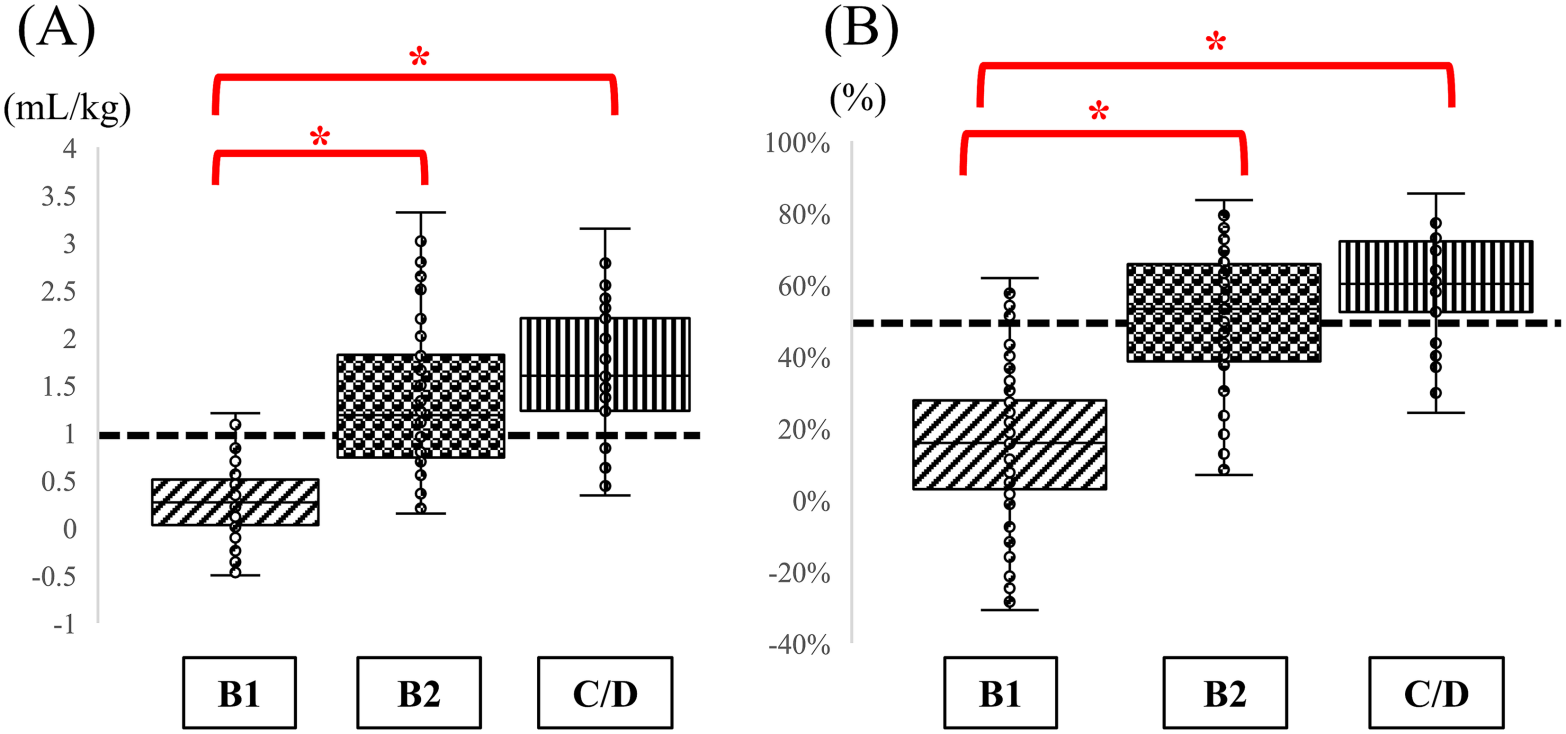
Box plots of quantitative regurgitant indices across ACVIM stages in dogs with MMVD. **(A)** Box plots of RVol/kg across ACVIM stages. The dashed horizontal line indicates the visual reference value of RVol/kg = 1.0 mL/kg. **(B)** Box plots of RF across ACVIM stages. The dashed horizontal line indicates the threshold used to define severe MR (RF = 50%). *p < 0.05 vs. stage B1. ACVIM, American College of Veterinary Internal Medicine; MMVD, myxomatous mitral valve disease; MR, mitral regurgitation; RF, regurgitant fraction; RVol/kg, body weight–normalized regurgitant volume.

Regurgitant indices are summarized in Table 1. RVol/kg and RF increased significantly with advancing ACVIM stage (both *p* < 0.001), with the highest values observed in stage C/D (Figure 1). *Post hoc* analysis demonstrated that both RVol/kg and RF were significantly higher in stages B2 and C/D than in stage B1 (both *p* < 0.001). However, no significant differences were observed between stages B2 and C/D. Most dogs in stages B2 and C/D showed RVol/kg values greater than 1.0 mL/kg, whereas severe MR defined by (RF ≥ 50%) was identified in 10.8% of dogs in stage B1, 56.1% in stage B2, and 77.8% in stage C/D (Figure 1). Overall, severe MR (RF ≥ 50%) was identified in 62 of 167 dogs (37.1%).

### 3.4 Correlation

Correlation analyses between regurgitant indices and conventional echocardiographic parameters are summarized in Table 2. RVol/kg and RF showed significant positive correlations with conventional echocardiographic variables, including the LA/Ao ratio, LVIDDN, and E-wave, whereas correlations with FS were weak (all *p* < 0.05). In particular, the mitral-aortic VTI ratio showed the strongest correlation with both RVol/kg and RF (both *r* = 0.75; *p* < 0.001).

**Table 2:** Correlations between quantitative regurgitant indices and conventional echocardiographic variables.

Variable	*r* with RVol/kg	*p*-value	*r* with RF	*p*-value
FS (%)	0.27	<0.01	0.22	<0.01
LA/Ao	0.71	<0.01	0.68	<0.01
LVIDDN	0.64	<0.01	0.57	<0.01
E-wave (m/s)	0.69	<0.01	0.65	<0.01
Mitral–aortic VTI ratio	0.75	<0.01	0.75	<0.01

E-wave, early diastolic transmitral velocity; FS, fractional shortening; LA/Ao, left atrial-to-aortic ratio; LVIDDN, body-weight–normalized left ventricular internal diameter in diastole; RF, regurgitant fraction; RVol/kg, regurgitant volume normalized to body weight; VTI, velocity-time integral.

### 3.5 Logistic regression analysis

The logistic regression analysis for identifying severe MR (RF ≥ 50%) is summarized in Table 3. The univariable logistic regression analysis identified several echocardiographic variables associated with severe MR. In the multivariable analysis, LVIDDN (odds ratio = 1.20; 95% CI, 1.04–1.40, *p* < 0.05) and the mitral-aortic VTI ratio (odds ratio = 1.45; 95% CI, 1.27–1.66, *p* < 0.01) remained independent predictors. No substantial multicollinearity was identified among the variables included in the multivariable analysis (all variance inflation factors < 2.0). Variable selection using Akaike information criterion-based stepwise procedures resulted in a final model including only the mitral-aortic VTI ratio and LVIDDN.

**Table 3:** Logistic regression analysis for identifying severe MR (RF ≥ 50%).

Variable	Univariable OR (95% CI)	*p*-value	Multivariable OR (95% CI)	*p*-value
FS (per 10% increase)	1.5 (1.1–2.0)	0.01	-	-
LA/Ao (per 0.1 increase)	1.3 (1.2–1.5)	<0.01	-	-
E-wave (per 10-cm/s increase)	1.7 (1.4–2.0)	<0.01	-	-
LVIDDN (per 0.1 increase)	1.4 (1.3–1.6)	<0.01	1.2 (1.0–1.4)	0.01
Mitral–aortic VTI ratio (per 0.1 increase)	1.5 (1.4–1.8)	<0.01	1.5 (1.3–1.7)	<0.01

CI, confidence intervals; E-wave, early diastolic transmitral velocity; FS, fractional shortening; LA/Ao, left atrial-to-aortic ratio; LVIDDN, body-weight–normalized left ventricular internal diameter in diastole; MR, mitral regurgitation; OR, odds ratio; RF, regurgitant fraction; VTI, velocity-time integral.

### 3.6 ROC analysis

**Figure 2:**
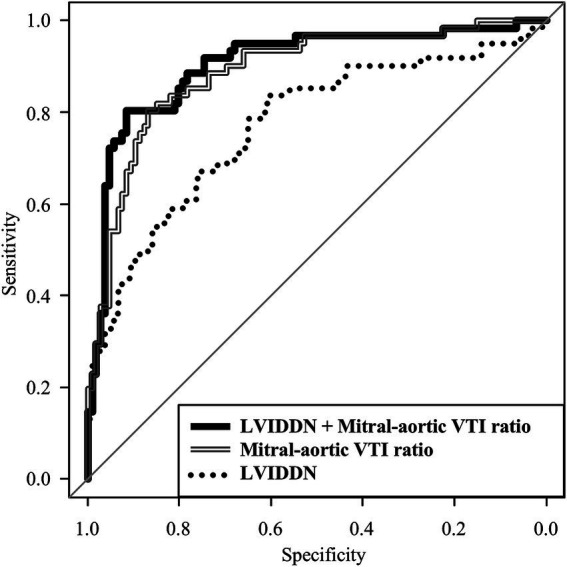
ROC curves for identifying severe mitral regurgitation (RF ≥ 50%) in dogs with MMVD. Solid black line, combined model (LVIDDN + mitral-aortic VTI ratio); gray line, mitral-to-aortic VTI ratio; dotted line, LVIDDN. LVIDDN, body-weight–normalized left ventricular internal diameter in diastole; MMVD, myxomatous mitral valve disease; RF, regurgitant fraction; ROC, receiver operating characteristic; VTI, velocity-time integral.

The results of the ROC analysis to identify severe MR (RF ≥ 50%) are summarized in Table 4. The ROC curves of the individual echocardiographic variables are shown in Supplementary Figure S2. Among the individual variables, the mitral-aortic VTI ratio showed the highest diagnostic performance (AUC = 0.89; 95% CI, 0.84–0.94). Among the conventional parameters, the LA/Ao ratio and E-wave showed moderate diagnostic performance (AUC = 0.84), whereas LVIDDN showed lower performance (AUC = 0.77), and FS showed the lowest performance (AUC = 0.62). The combination of LVIDDN and the mitral-aortic VTI ratio yielded an AUC of 0.91 (95% CI, 0.85–0.96) (Table 4 and Figure 2). However, the difference in AUC between the combined model and the mitral-aortic VTI ratio alone was not statistically significant according to DeLong’s test (*p* = 0.336).

**Table 4:** Diagnostic performance of echocardiographic variables for identifying severe MR (RF ≥ 50%).

Variable	AUC (95% CI)	Cutoff	Sensitivity (%)	Specificity (%)	PPV (%)	NPV (%)
Mitral–aortic VTI ratio	0.89 (0.84–0.94)	1.4	80	87	78	88
LA/Ao	0.84 (0.77–0.90)	1.7	80	77	67	87
E-wave (m/s)	0.84 (0.77–0.90)	1.0	71	84	55	87
LVIDDN	0.77 (0.69–0.85)	1.6	84	60	55	87
FS (%)	0.62 (0.54–0.70)	41.2	92	37	46	89
LVIDDN + mitral–aortic VTI ratio	0.91 (0.85–0.96)	0.46	92	80	84	88

For the combined model, the cutoff value represents the predicted probability derived from the logistic regression.

AUC, area under the curve; CI, confidence interval; E-wave, early diastolic transmitral velocity; FS, fractional shortening; LA/Ao, left atrium-to-aorta ratio; LVIDDN, body-weight–normalized left ventricular internal diameter in diastole; MR, mitral regurgitation; NPV, negative predictive value; PPV, positive predictive value; RF, regurgitant fraction; VTI, velocity-time integral.

## 4 Discussion

The present study demonstrated that quantitative regurgitant indices, particularly RVol/kg and RF, were significantly associated with conventional echocardiographic variables used in dogs with MMVD. However, discrepancies among conventional indices were observed, and severe MR defined by RF ≥ 50% was identified even in some dogs classified as ACVIM stage B1. These findings suggest that conventional staging systems and structural echocardiographic variables do not always fully reflect the underlying severity of MR. Quantitative regurgitant indices may therefore provide additional information regarding regurgitant burden beyond conventional remodeling-based assessment and may help identify dogs with substantial MR before overt cardiac remodeling becomes apparent.

Among the conventional variables, the LA/Ao ratio, LVIDDN, and E-wave showed significant correlations with RVol/kg and RF. A previous study demonstrated that these variables reflect the structural remodeling and altered filling dynamics associated with MR progression (2). The LA/Ao ratio is widely used as an indicator of chronic left atrial remodeling secondary to MR, whereas increased E-wave reflects elevated left atrial pressure and increased transmitral flow, commonly observed during MR progression (2, 5). However, because these variables primarily reflect secondary structural and hemodynamic changes associated with MR, they may not directly correspond to quantitative regurgitant severity itself (24). These characteristics may partly explain why their diagnostic performance is lower than that of the mitral-aortic VTI ratio.

FS showed weak correlations with RVol/kg and RF and demonstrated poor diagnostic performance for identifying severe MR. This finding was not unexpected because FS is strongly influenced by preload augmentation and compensatory hyperdynamic systolic function in dogs with chronic MR (25). In addition, systolic function may vary among dogs with advanced MMVD, with some dogs developing systolic dysfunction while others retain apparently normal systolic function. Therefore, FS alone may not accurately reflect the underlying MR severity in dogs with MMVD.

The mitral-aortic VTI ratio showed the strongest correlations with both RVol/kg and RF and demonstrated the highest diagnostic performance for identifying severe MR in the present population. However, these findings should be interpreted in the context of recent veterinary studies that have already evaluated similar relationships. A previous large-scale study demonstrated significant associations of the same mitral-aortic VTI ratio with RF, conventional echocardiographic variables, and ACVIM stage, and also reported its diagnostic performance for identifying severe MR (11). In addition, another study using the left ventricular early inflow-outflow index systematically examined the relationships between RF and conventional echocardiographic variables in dogs with MMVD (12). Therefore, the principal analyses of the present study, including the relationship between RF and conventional echocardiographic variables and the diagnostic performance of the mitral-aortic VTI ratio for severe MR, should not be interpreted as establishing these relationships *de novo*. Rather, the present findings provide supportive evidence that these previously reported relationships are also observed in a predominantly small-breed Japanese MMVD population. The dogs included in the present study were predominantly small-breed dogs, including Chihuahuas, Toy Poodles, Cavalier King Charles Spaniels, Pomeranians, and Miniature Dachshunds, and had median body weights of 5.5, 4.5, and 3.2 kg in stages B1, B2, and C/D, respectively. Because quantitative MR assessment and calculation of the mitral-aortic VTI ratio rely on measurements such as LVOT diameter and Doppler-derived VTI measurements, these assessments may be technically more challenging in very small-breed dogs. Therefore, validation in this population is clinically meaningful and represents the main contribution of the present exploratory study. Although the optimal cutoff identified in the present study (1.4) was slightly higher than the previously reported cutoff value of 1.27 (11), the difference was modest and may reflect differences in breed distribution, body size, disease-stage composition, and measurement variability inherent to retrospective studies. These findings suggest that the mitral-aortic VTI ratio may be applicable across different MMVD populations, while also highlighting the need for further prospective validation. In particular, a recent veterinary study comparing echocardiographic variables with cardiac computed tomography–derived regurgitant indices demonstrated that the mitral-aortic VTI ratio correlated strongly with both computed tomography-derived RVol and RF (26). These findings support the clinical utility of the mitral-aortic VTI ratio as a simple Doppler-derived surrogate for MR severity. As this index can be obtained rapidly using routinely performed Doppler echocardiography without geometric assumptions or complex volumetric calculations, it may represent a practical approach for hemodynamic assessment in dogs with MMVD. However, the interpretation of the correlation between the mitral-aortic VTI ratio and RF requires some consideration. Because RF was calculated from the modified Simpson’s method of disks-derived total left ventricular stroke volume and LVOT-derived forward stroke volume, whereas the mitral-aortic VTI ratio was calculated as transmitral inflow VTI/LVOT VTI, the two indices share the LVOT VTI component but are not mathematically identical. Therefore, the observed correlation may partly reflect mathematical coupling and should be interpreted with this consideration. However, the correlation is also biologically plausible because both indices reflect the hemodynamic consequences of MR.

In the multivariable analysis, LVIDDN and the mitral-aortic VTI ratio remained independent predictors of severe MR. This finding should be interpreted as a descriptive statistical observation within an exploratory retrospective analysis, rather than as confirmatory evidence. Although the combined model including LVIDDN and the mitral-aortic VTI ratio showed a slightly higher AUC than the mitral-aortic VTI ratio alone, the difference was not statistically significant. Therefore, the combined model should not be interpreted as demonstrating superior diagnostic performance. Instead, these findings suggest that structural remodeling and Doppler-derived assessment of regurgitant burden may provide complementary information regarding MR severity in dogs with MMVD.

The volumetric method used in this study was indirect and may have been affected by cumulative measurement variability. In particular, RF is derived from multiple measured and calculated variables and may amplify the measurement error. In addition, volumetric estimates obtained using the modified Simpson’s method of disks are influenced by image-plane selection, endocardial border tracing, and assumptions regarding ventricular geometry. The left apical four-chamber view was selected in accordance with established echocardiographic recommendations for volumetric assessment using the modified Simpson’s method of disks (21); however, different echocardiographic views may yield slightly different volume estimates, which could contribute to variability in calculated RVol and RF. These methodological limitations may also have influenced the classification of some dogs, including the identification of severe MR (RF ≥ 50%) in a subset of stage B1 dogs. Furthermore, occasional negative values of RVol or RF may occur because of cumulative measurement error and the propagation of variability across multiple derived measurements; therefore, such values should be interpreted cautiously. The modified Simpson’s method of disks may also underestimate ventricular volume in dogs with altered ventricular geometry (22, 27). Reference imaging modalities such as three-dimensional echocardiography, multidetector computed tomography, and cardiac magnetic resonance imaging may provide more comprehensive assessment of cardiac volume and regurgitant severity (26, 28, 29). Previous studies have demonstrated good agreement between three-dimensional echocardiography and multidetector computed tomography for assessment of left ventricular volume in dogs (28), whereas cardiac magnetic resonance imaging is widely used as an important reference modality for MR quantification in human medicine (29). However, these modalities remain less commonly applied in routine veterinary clinical practice, and comparative data in dogs with MMVD are still limited. Therefore, although volumetric assessment is not the definitive gold standard method, the quantitative regurgitant indices used in the present study may be considered clinically applicable surrogate measures of MR severity, provided that their methodological limitations are recognized.

Beyond the limitations of the volumetric method itself, this study has several additional limitations. First, this was a retrospective observational study, and no pre-specified primary endpoint or *a priori* hypothesis was established. Therefore, the findings should be interpreted as exploratory and require confirmation in prospective studies. In addition, treatment status was not standardized among the dogs. Many dogs, particularly those with a history of CHF, were receiving medical therapy, including pimobendan, diuretics, and angiotensin-converting enzyme inhibitors. These treatments may alter preload and loading conditions and potentially influence echocardiographic variables such as the E-wave, the LA/Ao ratio, and ventricular dimensions (30–32). Second, measurements were obtained from stored echocardiographic images, and the respiratory phase was retrospectively selected based on minimal thoracic motion. Third, comparisons with reference imaging modalities such as cardiac magnetic resonance imaging, cardiac computed tomography, and three-dimensional echocardiography were not performed in the present study. Finally, because the study population consisted primarily of dogs evaluated at referral institutions, a selection bias toward more advanced diseases may have been present.

In conclusion, quantitative regurgitant indices, including RVol/kg and RF, were significantly associated with conventional echocardiographic variables in dogs with MMVD, and they provided complementary information regarding MR severity in addition to conventional staging and structural assessment. The mitral-aortic VTI ratio showed strong correlations with quantitative regurgitant indices and demonstrated high diagnostic performance in identifying severe MR. Integration of quantitative regurgitant assessment with conventional echocardiographic evaluation may provide complementary information for the characterization of MR severity in dogs with MMVD, although prospective studies are required to confirm these findings.

## Data Availability

The original contributions presented in the study are included in the article/Supplementary material, further inquiries can be directed to the corresponding author/s.
