# Supplementary material for: Quantitative mitral regurgitation and conventional echocardiographic variables in canine myxomatous mitral valve disease
Source: Front Vet Sci. 2026 Jul 3;13:1885087. doi: 10.3389/fvets.2026.1885087 (PMC13375524; doi:10.3389/fvets.2026.1885087)
Supplement: Supplementary file 1 [file Data_Sheet_1.docx]

Supplementary Material

**Supplementary Table S1.** Breed distribution according to ACVIM stage in 167 dogs with MMVD.

| **Breed** | **B1** | **B2** | **C/D** |
| --- | --- | --- | --- |
| **Total** | **83** | **57** | **27** |
| **Chihuahua** | **14** | **16** | **2** |
| **Toy Poodle** | **15** | **6** | **6** |
| **Mixed-breed dog** | **8** | **8** | **0** |
| **Pomeranian** | **6** | **10** | **0** |
| **Cavalier King Charles Spaniel** | **6** | **8** | **3** |
| **Miniature Dachshund** | **7** | **3** | **4** |
| **Shih Tzu** | **5** | **1** | **4** |
| **Miniature Schnauzer** | **3** | **2** | **3** |
| **Maltese** | **1** | **2** | **5** |
| **Yorkshire Terrier** | **2** | **0** | **0** |
| **Italian Greyhound** | **2** | **0** | **0** |
| **Boston Terrier** | **2** | **0** | **0** |
| **Beagle** | **1** | **0** | **0** |
| **Bichon Frise** | **1** | **0** | **0** |
| **Welsh Corgi** | **1** | **0** | **0** |
| **Shikoku** | **1** | **0** | **0** |
| **Doberman** | **1** | **0** | **0** |
| **Borzoi** | **1** | **0** | **0** |
| **Papillon** | **0** | **1** | **0** |

ACVIM, American College of Veterinary Internal Medicine; MMVD, myxomatous mitral valve disease.

**Supplementary Table S2.** Proportion of dogs receiving pimobendan, angiotensin-converting enzyme inhibitors, and diuretics according to ACVIM stage.

| **Medication** | **B1 (n=83)** | **B2 (n=57)** | **C/D (n=27)** |
| --- | --- | --- | --- |
| **Pimobendan** | **4 (4.8%)** | **22 (38.6%)** | **27 (100%)** |
| **Angiotensin-converting enzyme inhibitor** | **17 (20.5%)** | **22 (38.6%)** | **26 (96.3%)** |
| **Diuretic** | **0 (0%)** | **6 (10.5%)** | **27 (100%)** |

ACVIM, American College of Veterinary Internal Medicine.

**Supplementary Figure S1.** Representative echocardiographic measurements used for calculation of quantitative regurgitant indices.

**
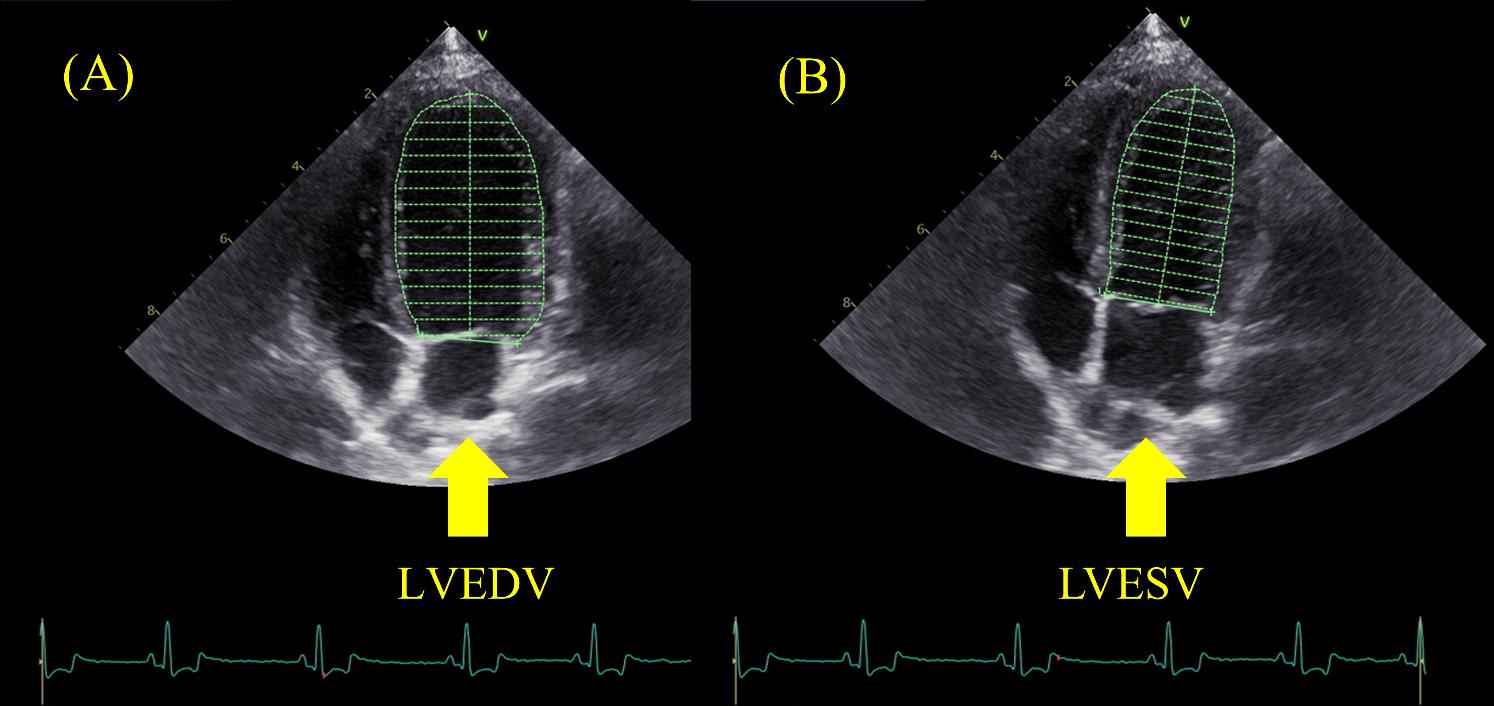
**

**
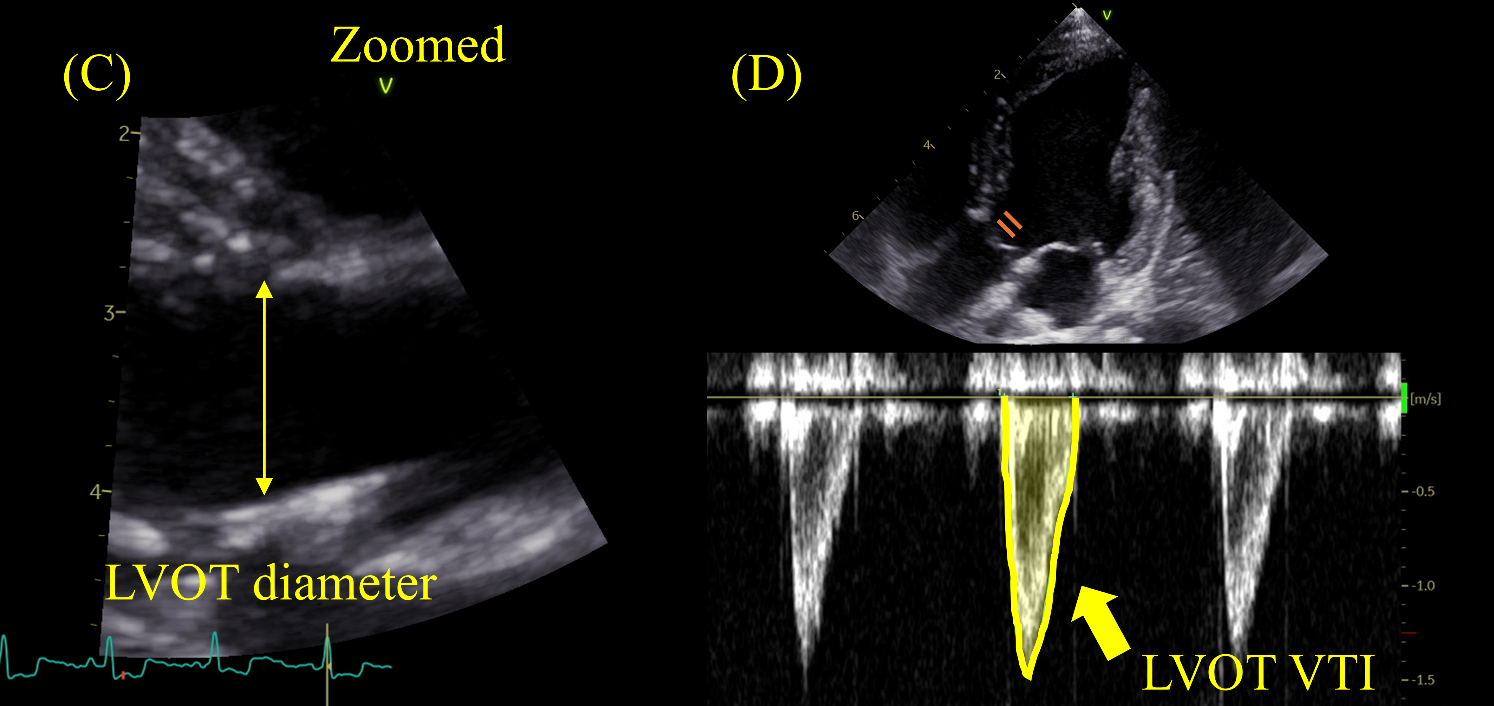
**

(A) LVEDV measured from the left apical four-chamber view using the modified Simpson’s method of disks.

(B) LVESV measured from the left apical four-chamber view using the modified Simpson’s method of disks.

(C) Measurement of LVOT diameter at the aortic annular level (hinge-point to hinge-point) from a zoomed right parasternal long-axis view at the frame corresponding to maximal opening of the aortic valve leaflets.

(D) Measurement of LVOT VTI obtained using pulsed-wave Doppler from the left apical five-chamber view. The orange annotation on the two-dimensional image indicates the sample volume position within the subaortic LVOT. The corresponding pulsed-wave Doppler tracing was used to measure LVOT VTI.

LVEDV, left ventricular end-diastolic volume; LVESV, left ventricular end-systolic volume; LVOT, left ventricular outflow tract; VTI, velocity-time integral.

**Supplementary Figure S2.** ROC curves of individual echocardiographic variables for identifying severe MR (RF ≥ 50%) in dogs with MMVD.


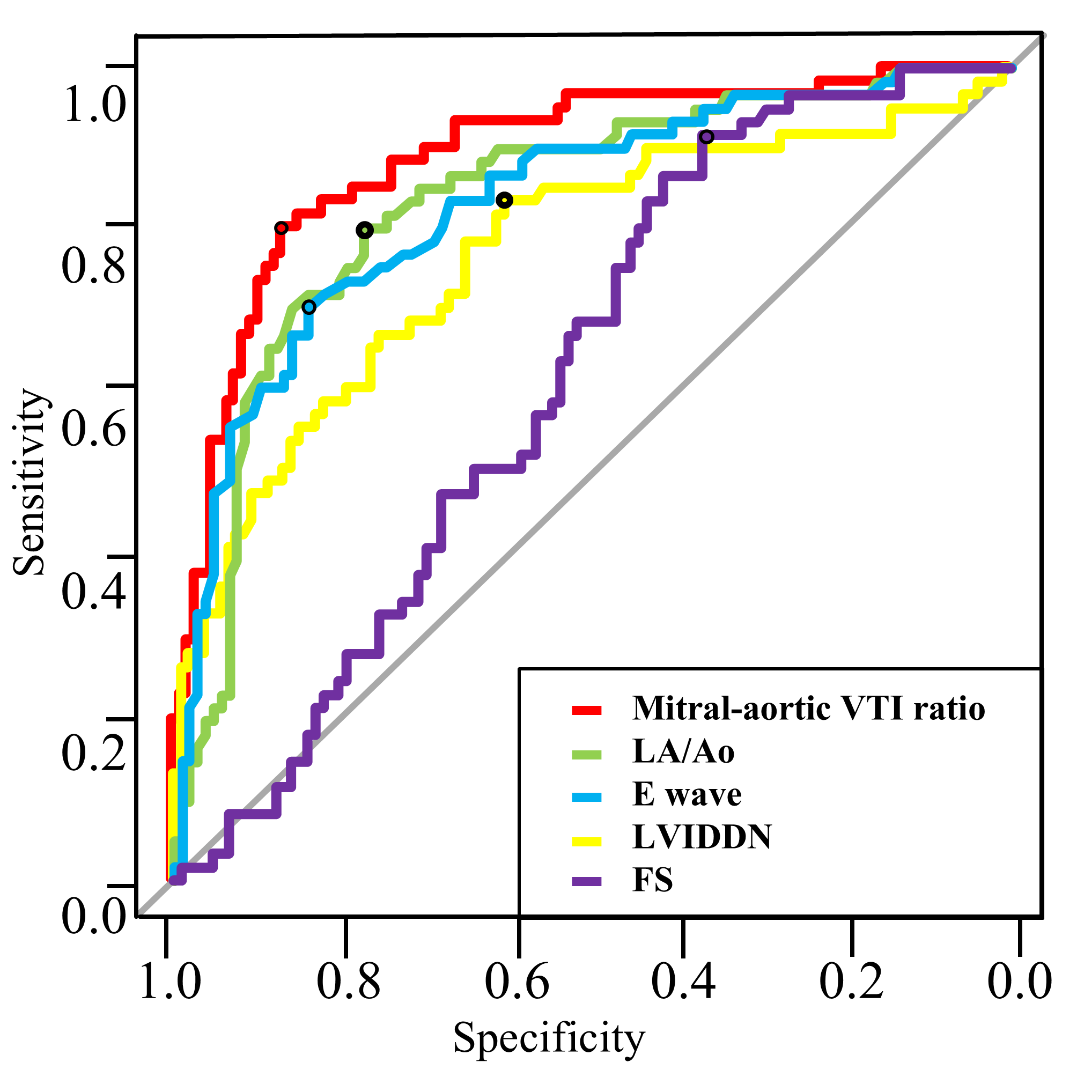


LA/Ao, left atrium-to-aorta ratio; LVIDDN, left ventricular internal diameter in diastole normalized to body weight; MMVD, myxomatous mitral valve disease; MR, mitral regurgitation; RF, regurgitant fraction; ROC, receiver operating characteristic; VTI, velocity-time integral.
